# Non-destructive monitoring of mouse embryo development and its qualitative evaluation at the molecular level using Raman spectroscopy

**DOI:** 10.1038/srep43942

**Published:** 2017-03-08

**Authors:** Mika Ishigaki, Kosuke Hashimoto, Hidetoshi Sato, Yukihiro Ozaki

**Affiliations:** 1 Department of Chemistry, School of Science and Technology, Kwansei Gakuin University, 2-1 Gakuen, Sanda, Hyogo 669-1337, Japan; 2 Department of Biomedical Chemistry, School of Science and Technology, Kwansei Gakuin University, 2-1 Gakuen, Sanda, Hyogo 669-1337, Japan

## Abstract

Current research focuses on embryonic development and quality not only by considering fundamental biology, but also by aiming to improve assisted reproduction technologies, such as *in vitro* fertilization. In this study, we explored the development of mouse embryo and its quality based on molecular information, obtained nondestructively using Raman spectroscopy. The detailed analysis of Raman spectra measured *in situ* during embryonic development revealed a temporary increase in protein content after fertilization. Proteins with a β-sheet structure—present in the early stages of embryonic development—are derived from maternal oocytes, while α-helical proteins are additionally generated by switching on a gene after fertilization. The transition from maternal to embryonic control during development can be non-destructively profiled, thus facilitating the *in situ* assessment of structural changes and component variation in proteins generated by metabolic activity. Furthermore, it was indicated that embryos with low-grade morphology had high concentrations of lipids and hydroxyapatite. This technique could be used for embryo quality testing in the future.

Fertilized ovum represents the origin of life. It exhibits totipotency and can create new life with genetic diversity. Several studies on embryonic development in cleavage stages have provided considerable information regarding pre-implantation RNA and protein variation patterns^1^,^2^. These studies have also suggested the secret of life by the detailed analysis of these material variations after fertilization. A mouse embryo traces some developmental stages during pre-implantation phase as follows: the embryos are in the pronuclear stage approximately half a day after fertilization, 2-celled stage after one and half days, and 8-celled stage after two and half days^3^. However, not all fertilized ova survive, and some of them just stop developing further. The material variations within the embryo, such as RNA and proteins, during the egg growth have been mainly investigated by the following methods: polypeptide analysis using embryonic RNA with sodium dodecyl sulfate-polyacrylamide gel electrophoresis (SDS-PAGE), specific amplification of RNA by real-time polymerase chain reaction (RT-PCR), and library construction of cDNA and screening^1^,^2^,^4^,^5^,^6^,^7^. It has been reported that an embryo primarily depends on the proteins and RNA synthesized and accumulated during oocyte growth until the middle 2-celled stage^2^,^7^. Furthermore, many genes are switched on as early as at the middle 2-celled stage^2^,^7^. Once genes are activated, the pattern of gene transcription established at the time of genomic activation in the 2-celled stage is perpetuated into the blastocyst and mRNA is continuously accumulated. The profile of the synthesis pattern of embryonic proteins proved that the periods of greatest changes for proteins are the late 1-celled and mid 2-celled stages^6^. The synthesis rates of almost 60% and 85% proteins, synthesized during 1- and 2-celled stages, respectively, change at least 2-fold and more. Furthermore, Giebelhaus *et al*. demonstrated that the transition from maternal to embryonic control of development can be expressed by the pattern of protein synthesis based on the quantitative histone analysis^7^. However, these studies on embryonic development based on the molecular levels of internal materials employed destructive analytical methods. Therefore, it is challenging to monitor *in situ* material variations in embryo at the molecular level.

The survival competence of embryos has received significant attention in several areas, such as aquaculture industry, infertility treatment for human. The survival potential is sometimes assessed using the key word “embryonic quality”. Embryonic quality is a crucial factor in assisted reproductive technology (ART) for infertility treatment. Human *in vitro* fertilization (IVF) was developed by Robert Edwards, and in 1978, the first baby using IVF was born^8^. ART has achieved remarkable progress and IVF has become one of the most important ARTs in fertility treatment.

In IVF treatment, during the incubation of embryos, its qualities are assessed and the best-quality embryos are transferred to the women’s womb. If the embryo gets implanted in the uterus and the pregnancy is confirmed, the IVF treatment is considered successful. The evaluation of embryonic quality is performed by visual inspection of cleavage rates and morphological feature of the ovum^9^,^10^,^11^,^12^,^13^. An ovum with uniform blastomeres, without fragmentation, is assessed as high grade, and has high potential for pregnancy^9^,^10^,^11^,^12^,^13^. In spite of human assistance, the pregnancy rate remains about 30% with treatment^14^. Morphological feature is a predictive factor of embryonic quality. However, the method seems to be empirical and phenomenalistic, and it does not specify the embryonic quality. Therefore, new evaluation techniques for embryonic quality are highly desired.

Several studies have reported alternate methods to assess the embryonic quality. In most cases, the variations in the materials in culture medium used for the embryonic incubation were examined and the embryonic quality was indirectly validated based on the metabolic activity of the embryo. For example, Conaghan *et al*. examined the relationship between the amount of pyruvic acid intake from the medium and the survival potential of human embryo^15^. They concluded that the embryos that take up a considerable amount of pyruvic acid have a high survival potential. Gardner *et al*. examined the relationship between the grade of embryo and the amount of glucose intake^16^. They reported that high grade embryo with good morphological feature takes up more glucose from the medium. Another study evaluated the metabolism in the culture medium by using near-infrared (NIR) and Raman spectroscopy^17^. The results of this study showed that the viability indices calculated by NIR or Raman spectroscopy were higher for the embryos that were implanted and resulted in a delivery, than those that failed to implant. However, we wanted to determine a more direct method to assess the embryonic quality, not only based on the morphological feature and culture medium, but also based on the molecular compositions obtained from the embryo non-destructively, non-invasively, and without labeling. For this purpose, Raman spectroscopy was expected to be one of the most powerful tools to monitor embryo development non-destructively at the molecular level.

Raman spectroscopy is one of the vibrational spectroscopies. Strong water bands in a Raman spectrum do not overlap with the fingerprint region (<2000 cm^−1^)^18^,^19^,^20^. Thus, Raman spectroscopy is much more suitable for biological applications than IR and NIR spectroscopies. Raman spectroscopy can provide rich molecular information nondestructively and *in situ*, without labeling. Even in complicated biological systems like embryos, information about biomaterials such as proteins, lipids, and DNA can be separately extracted from a Raman spectrum and their quantitative variations and structural changes can be investigated. Thus, biological and medical applications of Raman spectroscopy have received considerable interest from a number of researchers^18^,^19^,^20^.

There exist some articles reporting the biochemical composition of mouse oocyte studied by Raman spectroscopy and imaging^21^,^22^,^23^,^24^. For example, Davidson *et al*. investigated the cytoplasm biochemistry for the oocytes collected with different maturation methods and proved that mouse oocyte matured *in vitro* were protein-deficient compared to the ones grown *in vivo*^21^. Furthermore, Bogliolo *et al*. analyzed the link between aging and oxidative damage in mouse oocyte by Raman spectroscopy and concluded that the oxidative-damaged and old oocytes showed significant differences from young oocytes in the bands due to lipid and protein components^24^.

The present study, on the other hand, explored the dynamics of variations in biological components with the egg development by discussing about the protein-lipid relative concentration, secondary structural changes of proteins, and variations in the environments of tyrosine residues comprehensively, that occurred in conjunction with each other. Furthermore, it is also a feature of the present study to study the material variations by using Raman spectra obtained from several points per embryo. In the previous researches of mouse oocyte, Raman spectral data were collected in two-dimensional mapping mode or in line scan mode in order to smooth out the egg heterogeneity. It is true that eggs and embryos have heterogeneity of chemical components^22^. However, these methods are too much time consuming and unrealistic when we look into future medical applications of Raman spectroscopy to the assessment of embryonic development and its quality. If the egg development and its quality can be evaluated beyond the location dependency from the data measured from several points per embryo in a short time, it is considered that the possibility of practical application of Raman spectrum can be proved.

## Materials and Methods

### Mouse embryo

In this study, 5 stages of mouse eggs and embryos were used; unfertilized, pronuclear, 2-celled, 4-celled, and 8-celled stages of ICR mouse. These samples that had been frozen preserved by simple vitrification were purchased from Trans Genic Inc. (Japan). Eggs were obtained from the mice subjected to superovulation treatment and embryos were made by *in vitro* fertilization. After the *in vitro* fertilization was carried out, they were stored by simple vitrification. The detailed information about the protocols and reagents of all processes can be referred in refs 25 and 26. In order to recover the vitrified samples, they were immediately thawed with 900 μl 0.25 M sucrose medium preheated at 37 °C by pipetting 30 seconds after removal from a freeze storage tank with liquid nitrogen. The contents of the cryotube were transferred into a quartz petri dish. 400–500 μl 0.25 M sucrose was injected into the cryotube and the contents were transferred into a quartz dish again to dilute the cryoprotectant and to ensure that all of the embryos have been transferred. Soon after the warming, the samples in the quartz dish with sucrose media were measured in that state.

The experiments were performed in accordance with the fundamental guidelines for the proper conduct of animal experiments and related activities in academic research institutions under the jurisdiction of Ministry of Education, Culture, Sports, Science and Technology in Japan. The present study was approved by the ethics committee of Kwansei Gakuin University.

### Raman measurements and Multivariate analysis

The Raman spectrometer used was a micro Raman system (Nanofinder, Tokyo Instruments), which consists of a 785 nm Ti:sapphire laser (Mega-opt, Japan), a Raman spectrometer with a grading (600 1/mm, 750 nm brazed), and a Peltie cooled CCD detector (BRDD, Andor Technology). The objective lens used was a 40 times emersion type (OLYMPUS, UApoN 340, 40×/1.15 w) and the laser spot size was 1 μm laterally and 5μm axially. The laser power at the sample point was 30 mW, and the total exposure time was 30 s (15 s × 2). The system was equipped with a CO_2_ incubator, and the measurement was performed under the conditions of 5% CO_2_ and 37 °C.

In general, it is said that mammalian embryos are very sensitive to the light and it is desirable to keep the light exposure to a minimum^27^,^28^. Therefore, it was decided to measure 4–8 points per embryo at random and 3–6 embryos for each developmental stage. Therefore, the averaged Raman spectrum for each developmental stage reflected not only the embryonic heterogeneity but also the individual differences.

For the preprocessing of Raman spectra, the background noise due to the Raman system and spectral contributions from quartz dish and sucrose medium were subtracted at first, and the autofluorescence background from a sample was removed by 5^th^-order polynomial fitting. Then, the spectral intensity was normalized with a standard band at 1004 cm^−1^ due to phenylalanine. The preprocessed Raman data were used for the calculation of averaged Raman spectra and multivariate analysis such as principal component analysis (PCA). The PCA was performed with chemometrics software Unscrambler X 10.3 (CAMO, Japan).

## Results and Discussion

### Changes in the Concentrations and Structures of Proteins During Embryonic Development

**Figure 1:**
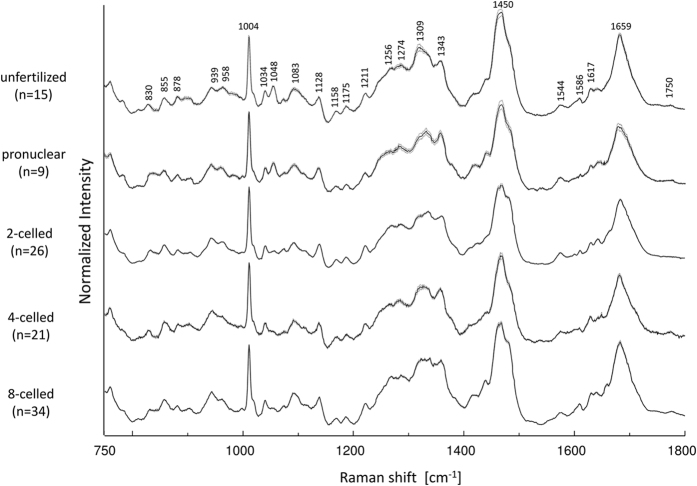
Averaged Raman spectra for five different developmental stages (unfertilized, pronuclear, 2-celled, 4-celled, and 8-celled) with ±1 standard error.

Figure 1 shows the averaged Raman spectra in the 750–1800 cm^−1^ region in mouse embryos in the 5 stages of embryo development: unfertilized (n = 15), pronuclear (n = 9), 2-celled (n = 26), 4-celled (n = 21), and 8-celled (n = 33) stages, with ± 1 standard error. The Raman data were obtained by selecting embryos assessed to have high-grade morphological features. Some characteristic bands from proteins, lipids, and DNA/RNA were clearly observed (Fig. 1). From the Raman spectra, molecular information at the different developmental stages of the embryo can be extracted non-destructively. Some prominent Raman bands that were assigned based on earlier reports^29^,^30^,^31^ are shown in Table 1.

**Figure 2:**
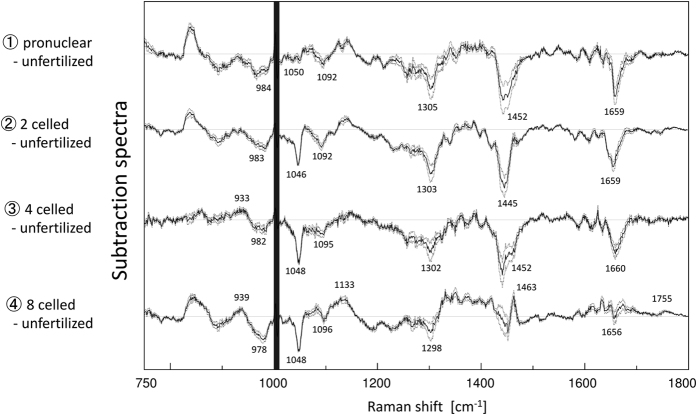
Difference spectra calculated by subtracting the unfertilized spectrum from the spectrum of each developmental stage with ±1 standard error.

Figure 2 shows the difference spectra calculated by subtracting the spectrum of the unfertilized stage from each spectrum. Some characteristic bands of proteins, lipids, and DNA were observed. Two minus bands around 1048 cm^−1^ and 1096 cm^−1^ in the difference spectra are assigned to PO_4_^3−^ and PO_2_^−^ backbone stretching modes of DNA^29^,^30^,^31^,^32^. They indicate that the relative concentration of DNA to phenylalanine (intensity of a band at 1004 cm^−1^ due to C-C symmetric ring breathing mode of phenylalanine was used as an internal standard of peak intensity) decreases after fertilization^29^,^30^ (the intensity decrease at 1048 cm^−1^ is also clear in the raw spectra; see Fig. 1). Bands at 1300 cm^−1^ and 1463 cm^−1^ are due to the CH_2_ twisting and CH_3_/CH_2_ deformation modes in lipids, respectively, and those at 1660 cm^−1^ and 1755 cm^−1^ arise from the C=C and C=O stretching modes, respectively, of lipids^29^,^30^,^31^. Note that the bands at 1660 cm^−1^ and 1463 cm^−1^ are overlapped with those from amide I and CH_3_/CH_2_ deformation modes in proteins. The change in relative intensity of the band at 1300 cm^−1^ in Fig. 2 suggests that the relative concentration of lipids to phenylalanine decreases immediately after fertilization and subsequently recovers with the development of the embryo. Furthermore, bands at around 940 cm^−1^ and 980 cm^−1^ assigned to C-C backbone stretching mode with α-helix and β-sheet structures of proteins, respectively^29^,^30^,^31^, were also observed in the difference spectra (Fig. 2).

**Figure 3:**
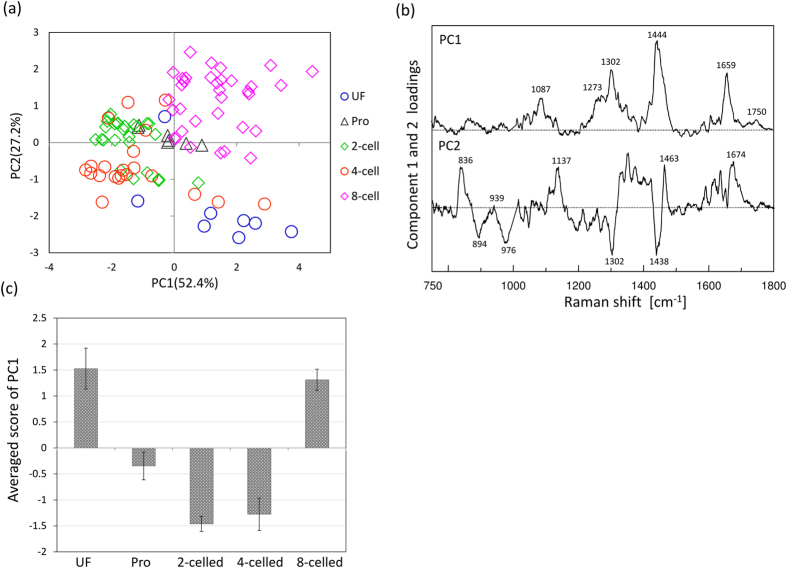
(**a**) Score plot of PCA performed for datasets of all developmental stages. (**b**) Loading plots of PC1 and PC2 for PCA in (**a**). (**c**) Averaged score of PC1 with the standard error.

In order to gain information on some characteristic components varying with the embryonic development, PCA was performed for the datasets of all the developmental stages. Figure 3(a) illustrates principal component (PC)1 and PC2 score plots of PCA, developed by using the 5 datasets. The score plots are classified depending on each developmental stage. Furthermore, unfertilized and 8-celled stages are clearly separated by PC2 even though these two groups have similar PC1. In the loading plot of PC1 shown in Fig. 3(b), peaks at 1087, 1273, 1302, 1444, 1659, and 1750 cm^−1^; they can be assigned to C-C stretching of alkyl chain, C=C groups in unsaturated fatty acids, CH_2_ twisting, CH_2_ bending and C=C stretching modes are attributed to lipids. With embryonic development, the score average of PC1 changes in a round trip, as shown in Fig. 3(c). This result also indicates that the relative concentration of lipids to phenylalanine varies in a cyclic manner with the embryonic development. The first possibility to explain the cyclic lipid variation is that the lipid concentration itself changes in a cyclic manner. Another possibility is that the concentration of proteins characterized by phenylalanine temporarily increases after fertilization due to the vital activity. By comprehensively assessing the subtraction results of the averaged spectra (as shown in Fig. 2), in which the intensities of many bands assigned to DNA (1048 cm^−1^ and 1090 cm^−1^) and lipids (1300 cm^−1^ and 1750 cm^−1^) become weaker during the stages after fertilization, the latter possibility that the protein levels temporarily increase may be more indicative of the actual reaction in the living embryo.

It is well known that the cytoplasm of oocyte has a complex cytoskeleton, including actin, turbulin, and cytokeratin^4^,^5^,^33^. The hypothesis that protein levels temporarily increase after fertilization, as mentioned earlier, is consistent with the report by Latham *et al*. who showed the profile of the synthesis pattern of embryonic proteins by two-dimensional gel protein database and proved that the maximal changes for proteins occur during the late 1-celled and mid 2-celled stages^6^. The synthesis rates of almost 60% and 85% proteins, which are synthesized during 1- and 2-celled stages, respectively, change by at least 2-fold and sometimes, greater. Furthermore, Giebelhaus *et al*. proved that actin and histone H3 mRNA present in oocyte almost disappear until 2-celled stage, using recombinant DNA probes having sequence homology to these mRNA species^7^. Thus, the transition from maternal to embryonic control of development can be expressed by the pattern of protein synthesis.

**Figure 4:**
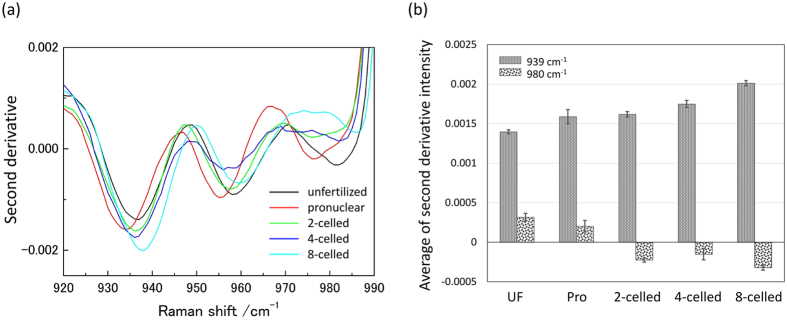
(**a**) Second derivative spectra of different developmental stages in the 910–980 cm^−1^ region. (**b**) The average of second derivative intensity with the sign inverse at 939 cm^−1^ and 980 cm^−1^ due to α-helix and β-sheet structure, respectively with the standard error.

As for the PC2 component in Fig. 3(a), the unfertilized and fertilized (pronuclear, 2-celled, 4-celled, and 8-celled stages) groups could be classified. In the loading plot of PC2, the peaks at 939 cm^−1^ and 980 cm^−1^, relating with the secondary structure of proteins were observed. In order to investigate secondary structure changes of proteins within the embryo, the wavenumber region between 910–980 cm^−1^ was used. The bands at around 940 cm^−1^ and 980 cm^−1^ are assigned to C-C backbone stretching modes of α-helix and β-sheet, respectively^29^,^30^,^31^. These bands overlapped with each other and also with other bands. In order to separate overlapped bands into more distinct components existing in the 910–980 cm^−1^ region, second derivative spectra were calculated, as shown in Fig. 4(a). Figure 4(a) demonstrates that the directions of intensity changes in the α-helix (939 cm^−1^) and β-sheet (980 cm^−1^) bands are opposite to each other with embryonic development; the band intensity at 939 cm^−1^ becomes stronger, while that at 980 cm^−1^ becomes weak with embryonic development. Figure 4(b) illustrates the average of second derivative intensity with the sign inverse at 939 cm^−1^ and 980 cm^−1^ due to the α-helix and β-sheet structure of proteins, respectively. The band from the β-sheet structure cannot be observed after 2-celled stages. Hence, it is very likely that the proteins with β-sheet structure, existing in the unfertilized and pronuclear stages, originate from maternal oocyte, and the proteins with the α-helix structure are generated by switching a gene on after the fertilization. This inference of the secondary structural changes of proteins is also consistent with the results obtained *in vitro* by other researchers^6^,^7^.

There are other bands characteristic of the secondary structure of proteins in the regions of amide I (1630–1680 cm^−1^) and amide III (1220–1285 cm^−1^)^29^,^30^,^31^. However, the protein bands characteristic for the protein secondary structure in these regions overlap with other bands from lipids and the protein in a complex manner. In contrast, there is only one band at 960 cm^−1^ due to hydroxyapatite in the region of 910–980 cm^−1^ used for the discussion of the secondary structural changes of proteins mentioned earlier. Thus, our wavenumber selection is useful for the analysis of the protein secondary structures.

**Figure 5:**
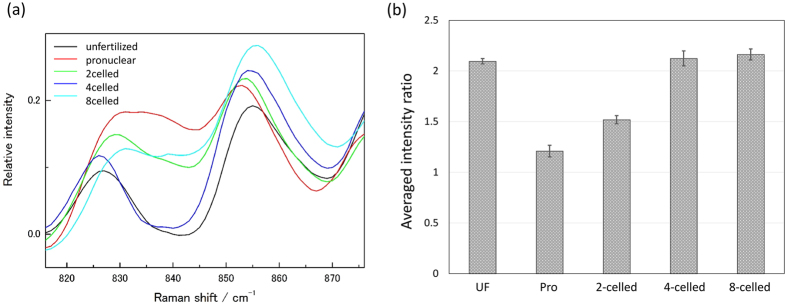
(**a**) Raman spectra in the region of tyrosine doublet in the different developmental stages. (**b**) The averaged intensity ratio for the tyrosine doublet 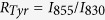 with the standard error for the five stages.

Next, the tyrosine doublet at 830 cm^−1^ and 855 cm^−1^, shown in Fig. 5(a), is discussed^29^,^30^,^31^. These two bands are due to Fermi resonance arising from the ring-breathing vibration and the overtone of an out-of-plane ring-bending vibration of the para-substituted benzene. The intensity ratio of these bands (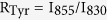) reflects the state of hydrogen bonding and the ionic state of hydroxyl group of tyrosine residue; while there are several tyrosine residues in the proteins, the Raman spectra can detect only the average state of the tyrosine residues^34^. When the hydroxyl group is held together with a strong hydrogen-bond acceptor and H_2_O with moderate strength by hydrogen-bonding, R_Tyr_ takes the value of nearly 0.5 and 1.2, respectively. When the hydroxyl group acts as a strong hydrogen-bond acceptor and it ionizes, 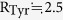 and 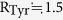, respectively. It is well known that hydrogen bonds act as a driving force to form secondary structures of a protein. Therefore, it is very interesting to bring a change in the relative intensity of the tyrosine doublet into the secondary structural information to interpret the activation of vital activity and embryonic development.

The intensity ratio R_Tyr_ is plotted versus developmental stages in Fig. 5(b). It was noted that the ratio changes with the embryonic development, and the changes in the ratio shown in Fig. 5(a) and (b) indicate that the hydroxyl group is in the ionic state immediately after the fertilization, and then changes to a strong hydrogen bound acceptor. It is likely that the protein secondary structural changes go through the intermediate state of ionic O-H with the protein synthesis during 1- and 2-celled stages, as mentioned earlier.

Based on the result that protein level temporary increased after fertilization, as mentioned earlier (Fig. 2), the secondary structural changes of proteins and the variations in tyrosine doublet suggest the following: the vital activity is initiated by fertilization and increased protein content is temporarily generated, especially from late 1-celled to mid 2-celled stages. The relative concentration of proteins comprising the original β-sheet structure in oocyte decreases and the result of tyrosine doublet shows that the O-H state is ionic. After that, the relative concentration of proteins with α-helical structure increases and the tyrosine residues assume that the O-H state is a strong hydrogen-bound acceptor. Therefore, the protein structures seem to be altered to the α-helix-rich and β-sheet-poor conformation.

In this manner, Raman spectroscopy can monitor the embryonic development nondestructively, and profile the variations in internal materials, such as material concentrations, secondary structural changes of proteins, and the side chain variations of proteins.

### Raman *in vivo* Monitoring of Embryonic Quality

**Figure 6:**
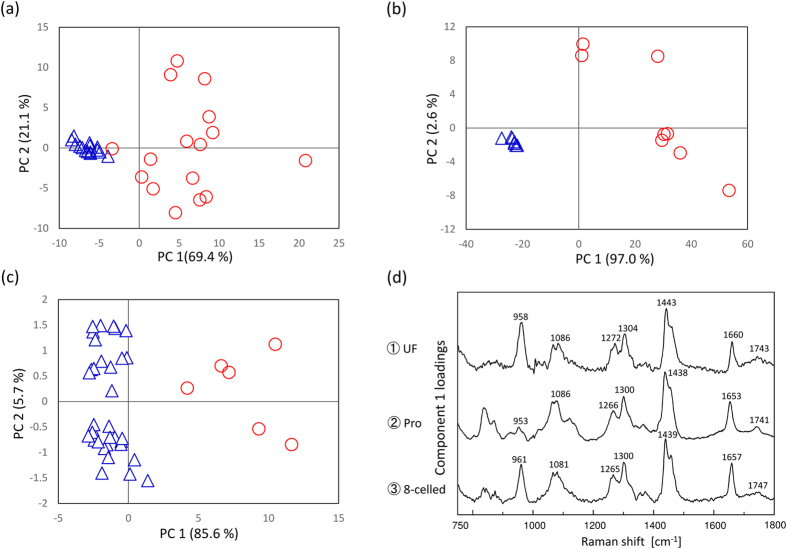
PCA score plots of (**a**) unfertilized, (**b**) pronuclear, and (**c**) 8-celled stages. (**d**) Loading plots of PC1 for the three different developmental stages of embryo.

**Figure 7:**
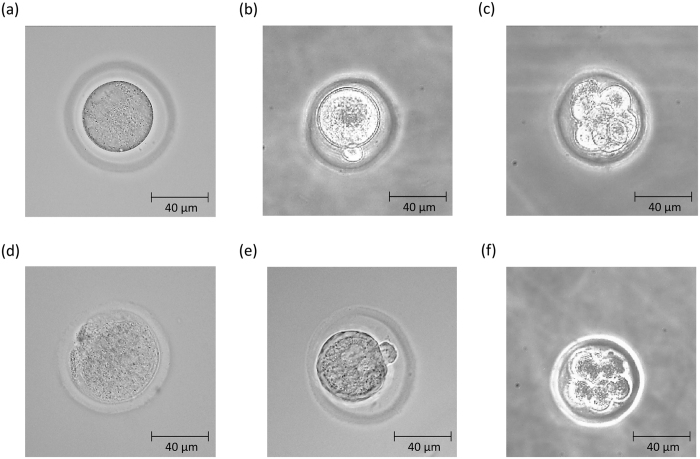
Examples of embryos (**a**–**c**) with high-grade morphological features in unfertilized, pronuclear, and 8-celled stages and (**d**–**f**) with low-grade morphology.

**Figure 8:**
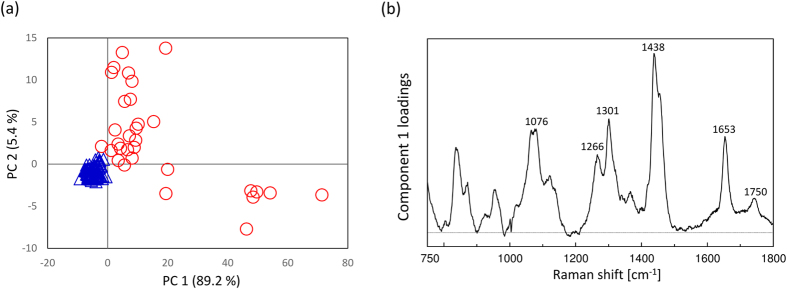
(**a**) PCA score plot of all data, including all developmental stages. (**b**) Loading plot of PC1.

In order to extract some characteristic components depending on the embryonic quality, PCA was performed for the dataset of each developmental stage (unfertilized (n = 31), pronuclear (n = 17), 2-celled (n = 26), 4-celled (n = 21), and 8-celled (n = 39) stages), including the dataset for the embryos with low-grade morphology. In the score plots of unfertilized, pronuclear, and 8-celled stages, the data are classified into two groups by PC1 as shown in Fig. 6(a–c). Figure 6(d) exhibits loading plots of PC1 for all the PCA in Fig. 6(a–c). They demonstrate the separation factor of the data set into two groups. In Fig. 6(d), it is important to note that the 3 loadings plots have common peaks at 1085, 1270, 1300, 1440, 1660, and 1745 cm^−1^ from lipids which were common with the ones in the loading plot of PC1 as shown in Fig. 3(b) mentioned above, and another common peak at 960 cm^−1^ due to symmetric stretching of hydroxyapatite^18^,^19^,^35^. This implies that the concentrations of lipids and hydroxyapatite may be different between these two groups. When all data points in each score plot and morphological features were evaluated one by one, it became clear that the spectral data were grouped into two depending on morphology; the blue colored group has high grade morphological features and the red colored group has low grade morphology. Figure 7 displays the examples of the embryos with high (a–c) and low (d–f) grade morphological features in unfertilized, pronuclear, and 8-celled stages, respectively. The results shown in Figs 6 and 7 indicate that embryos with low-grade morphological feature have high concentrations of lipids and hydroxyapatite. Furthermore, in the loading plot of PC2 for these 3 stages, many characteristic bands from proteins can be observed. The concentration variations depending on the positions in embryos with high grade morphological features are expected to be small, but large with low grade morphological features in the unfertilized and pronuclear stages, as shown in Fig. 6(a) and (b). On the other hand, at the 8-celled stage in Fig. 6(c), the protein concentration differences depending on each location within the embryos are large even though they have high-grade morphology. With further development, the compositions and concentrations of proteins may vary. Similarly, PCA analysis was also performed using the Raman spectral data set including all developmental stages (unfertilized (n = 31), pronuclear (n = 17), 2-celled (n = 26), 4-celled (n = 21), and 8-celled (n = 39) stages) as shown in Fig. 8(a). This data set is also classified into two groups by PC1. Figure 8(b) exhibits the loading plot of PC1 for the PCA shown in Fig. 8(a). Note that in the loading plot, some lipids bands also appear at 1076, 1266, 1301, 1438, 1653, and 1750 cm^−1^. This indicates that the concentrations of lipid components are high in the mouse embryos with low-grade morphological features. Moreover, the compositional differences related to the morphological features are larger than the ones between the developmental stages. Here, the morphological differences are expected to reflect the embryonic quality and viability. Therefore, the embryos with low quality may be distinguished by high lipid concentrations.

It is well known that lipids are a potent energy source for embryonic development^36^,^37^,^38^. There are some reports investigating the role of lipid contents as influencing factors for embryonic quality. Abd *et al*. proved that embryos produced *in vitro* have higher lipid content compared with the ones produced *in vivo*^39^. Dunning *et al*. investigated the metabolic pathway generating cellular energy from lipids responsible for the oocyte developmental competence and early embryonic development, by assessing β-oxidation of fatty acids. They concluded that upregulation of β-oxidation caused by the supplementation of L-carnitine improved the development competence of embryos^37^. Hence, fatty acids are important energy source for oocyte and embryo development, and they should be appropriately metabolized for the proper growth of oocyte and embryo. From their perspective of the relationship between lipid metabolism and embryonic survival potential, our results of high lipid concentration can be interpreted by assuming that embryos lost their viability competence by metabolic abnormality. However, thus far, little is known about the lipid metabolism in embryo and the relationship between lipid contents and embryonic quality^37^,^38^,^39^.

In cell biology, hydroxyapatite is sometimes closely related to cell death and cell abnormality^35^. For instance, the connection between cell death and cell calcification has often been investigated in vascular and muscle cells^40^,^41^,^42^. Proudfoot *et al*. showed that apoptosis initiates vascular calcification^43^. Furthermore, many reports were published showing that the calcification of cells and tissues is strongly associated with malignant disease. Morgans *et al*. proved that hydroxyapatite facilitates breast cancer progression by exerting biological effects on surrounding cells^44^. In the analysis of human brain tissue by Raman spectroscopy, a strong peak at 960 cm^−1^ due to hydroxyapatite was reported in the abnormal tissue. The detection of a strong band due to hydroxyapatite for embryos with low-grade morphology indicates that the embryos are either dead or abnormal. The result indicates that the band due to hydroxyapatite at 960 cm^−1^ can be an effective distinguishing factor for embryonic quality.

Thus, our results demonstrate the possibility of evaluating embryonic quality by using lipid and hydroxyapatite bands: the lipid and hydroxyapatite bands can be marker bands in assessing the embryonic quality.

In this way, embryonic development and its quality were investigated by use of Raman spectroscopy. The results showed the secondary structural changes of proteins, changes in the tyrosine environments, and the concentration variations of some biomaterials depending on the egg development and its quality. However, it is necessary to examine the credibility of these results from the relevance to egg heterogeneity. By using Raman spectroscopic mapping of fixed samples, Davidson *et al*. proved that mouse oocyte and early embryo had biochemical variations depending on the location within them^22^. They calculated the averaged Raman spectra using big spectral dataset obtained by mappimg mode and average out the heterogeneity. In the present study, on the other hand, Raman spectra were obtained from several points per embryo by the point mode. The measurement conditions were decided to reduce the phototoxicity for the embryos by causing light exposure because fresh samples, not fixed, were investigated in the present study.

Then, the variances from local dependence and different developmental stages should be quantitatively argued. To analyze the spectral variances due to local dependences at first, an averaged Raman spectrum (I_ij_) and standard error (σ_ij_) for one embryo were calculated from several Raman spectra obtained from the embryo by the point mode, where i and j were indices expressing the number of embryos and the wavenumber of a Raman shift, respectively. The variance from heterogeneity was defied as the average of the fraction 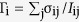, where the indices j were eliminated in the case that I_ij_ < 0.1 to escape the extreme problem. The averaged Γ_i_ for high-grade became (4.7 ± 0.6) × 10^−2^ and for low-grade was (13.4 ± 1.4) × 10^−2^. The result indicated that high-grade and low-grade embryos had total spectral variance about 5% and 10%, respectively due to heterogeneity. By comparing these two values, it may be concluded that the spectral variances are an indicator for the assessment of embryonic quality.

Furthermore, spectral variances between developmental stages were calculated. By using the standard error for the spectral variance (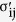) from averaged Raman spectra on the last developmental stage, the variances between developmental stages were defined as 
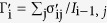
. Here, i and j represent the indices of developmental stage and wavenumber of Raman shift, respectively, and the summation for index j was performed only for j which satisfies the condition 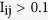 as mentioned above. It becomes as (6.5 ± 1.2) × 10^−2^. It takes the value in the middle of the ones due to heterogeneity between high-grade and low-grade embryo. Therefore, the material variations between developmental stages can be discussed in the group including only high-grade embryos.

In fact, several spectral data obtained from one embryos were classified into the same group of PCA score plots as shown in Figs 3(a), 6(a)~(c) and 8(a). The results indicate the bigger differences due to individual differences, developmental stages, and morphological features can be extracted excess beyond the egg heterogeneity by using multivariate analysis. It shows that the evaluation of egg development and its quality can be performed by using several Raman spectra measured from several points per embryo. This result may provide very useful information when considering practical assessment of the embryonic development and its quality.

## Conclusion

This study demonstrated the possibility for the *in situ* monitoring of embryonic development and assessment of embryonic quality in a nondestructive manner. We successfully measured *in situ* the Raman spectra obtained for unfertilized, pronuclear, 2-celled, 4-celled, and 8-celled developmental stages. The variations in proteins and lipids and the structural changes in proteins concomitant with embryo development and morphology were observed nondestructively. It has been suggested that the active generation of proteins with α-helix secondary structures occurs temporarily after fertilization. The protein production and secondary structural changes can be used as biomarkers to evaluate the activation of vital activity and viability. Our results also indicated that the changes in internal materials during the transition from maternal to embryonic control can be roughly delineated between the 2-celled and 4-celled stages. Furthermore, it is noteworthy that hydrogen-bound indices derived from the ratio of tyrosine doublet can be consistently interpreted with the corresponding changes in protein secondary structure.

Embryos with low-grade morphological feature have high concentrations of lipids and hydroxyapatite, indicating that the bands attributable to these components can be distinctive factors for the assessment of embryo quality.

It is essential to carefully examine the invasiveness of laser irradiation in embryos; this technique may be a novel and feasible method for noninvasive monitoring of embryonic development and testing its quality in the future. This technique is expected to be the first step in improving the success rate of IVF with embryonic assessment based on the molecular information.

## Additional Information

**How to cite this article:** Ishigaki, M. *et al*. Non-destructive monitoring of mouse embryo development and its qualitative evaluation at the molecular level using Raman spectroscopy. *Sci. Rep.* **7**, 43942; doi: 10.1038/srep43942 (2017).

**Publisher's note:** Springer Nature remains neutral with regard to jurisdictional claims in published maps and institutional affiliations.

## Figures and Tables

**Table 1:** Peak assignment in the Raman spectra from mouse embryos^29^,^30^,^31^,^32^.

Peak (cm^−1^)	DNA/RNA	Proteins	Lipids	Carbohydrates	Others
826	O-P-O str DNA/RNA	ring br Tyr			
855		ring br Tyr			
878		Try	C-C-N^+^ sym str	C-O-C ring	
939		C-C BK str α-helix			
958	sym str PO_4_^3−^				hydroxyapatite
980		C-C BK str β-sheet			
1004		sym ring br Phe			
1034		Phe			
1048	sym str PO_4_^3−^				
1076	sym str PO_4_^3−^		C-C		
1083		C-N str			
1096	sym str PO_2_^−^				
1128		C-N str		C-O str	
1158		C-C/C-N str			
1175	C, G	Tyr, Phe, C-H bend			
1211		C-C_6_H_5_ str Tyr, Phe			
1250–1275	A, T	Amide III	CH_2_ def		
1300			CH_2_ twi		
1309		CH_3_/CH_2_ twi, ben	CH_3_/CH_2_ twi, ben		
1343	G	CH def		CH def	
1450		CH def	CH def	CH def	
1463		CH_3_/CH_2_ def	CH_3_/CH_2_ def		
1544		Amide II			
1586		C=C Phe			
1617		C=C Phe, Tyr			
1659		Amide I	C=C str		
1750			C=O str		
